# Artificial intelligence-based locoregional markers of brain peritumoral microenvironment

**DOI:** 10.1038/s41598-022-26448-9

**Published:** 2023-01-18

**Authors:** Zahra Riahi Samani, Drew Parker, Hamed Akbari, Ronald L. Wolf, Steven Brem, Spyridon Bakas, Ragini Verma

**Affiliations:** 1 Diffusion & Connectomics In Precision Healthcare Research (DiCIPHR) Lab, University of Pennsylvania, Philadelphia, PA 19104 USA; 2 Center for Biomedical Image Computing and Analytics (CBICA), University of Pennsylvania, Philadelphia, PA 19104 USA; 3 Department of Radiology, Perelman School of Medicine, University of Pennsylvania, Philadelphia, PA 19104 USA; 4 Department of Neurosurgery, Perelman School of Medicine, University of Pennsylvania, Philadelphia, PA 19104 USA; 5 Department of Pathology & Laboratory Medicine, Perelman School of Medicine, University of Pennsylvania, Philadelphia, PA 19104 USA

**Keywords:** Computational models, Machine learning, Biomedical engineering

## Abstract

In malignant primary brain tumors, cancer cells infiltrate into the peritumoral brain structures which results in inevitable recurrence. Quantitative assessment of infiltrative heterogeneity in the peritumoral region, the area where biopsy or resection can be hazardous, is important for clinical decision making. Here, we derive a novel set of Artificial intelligence (AI)-based markers capturing the heterogeneity of tumor infiltration, by characterizing free water movement restriction in the peritumoral region using Diffusion Tensor Imaging (DTI)-based free water volume fraction maps. We leverage the differences in the peritumoral region of metastasis and glioblastomas, the former consisting of vasogenic versus the latter containing infiltrative edema, to extract a voxel-wise deep learning-based peritumoral microenvironment index (PMI). Descriptive characteristics of locoregional hubs of uniformly high PMI values are then extracted as AI-based markers to capture distinct aspects of infiltrative heterogeneity. The proposed markers are utilized to stratify patients’ survival and *IDH1* mutation status on a population of 275 adult-type diffuse gliomas (CNS WHO grade 4). Our results show significant differences in the proposed markers between patients with different overall survival and *IDH1* mutation status (*t* test, Wilcoxon rank sum test, linear regression; *p* < 0.01). Clustering of patients using the proposed markers reveals distinct survival groups (logrank; *p* < 10^−5^, Cox hazard ratio = 1.82; *p* < 0.005). Our findings provide a panel of markers as surrogates of infiltration that might capture novel insight about underlying biology of peritumoral microstructural heterogeneity, providing potential biomarkers of prognosis pertaining to survival and molecular stratification, with applicability in clinical decision making.

## Introduction

The tumor burden of diffuse gliomas extends beyond the radiographically visible border of tumor margin^1–3^. However, current clinical practice considers the tumor core (T1 contrast enhancing boundary) as the primary target of treatment^4,5^; therefore, infiltrative cancer in the peritumoral region (peritumoral T2 hyperintense tissue) remains un-resected which may result in tumor recurrence. Obtaining tissue biopsies can be hazardous in the peritumoral region due to the possibility of functional deficit^4,5^. As a result, characterization of the infiltrative heterogeneity in the peritumoral region is a critical need to inform clinical decision making. Previous attempts to characterize tumor infiltration applied various imaging modalities using manually delineated or heuristic-based labeling of infiltrative tissue^6–12^, but approaches using Diffusion Tensor Imaging (DTI) have been limited to clinically used measures^7–10^.

DTI is the Magnetic Resonance Imaging (MRI) modality that provides insight into tissue microstructure by measuring water diffusivity. In particular, the free water volume fraction, a measure of the amount of extracellular water^13^, is able to capture differences between infiltrative and vasogenic peritumoral regions by exploiting the variation in water movement restriction^14–16^. The overarching goal of this paper is to leverage this unique information of DTI and derive artificial intelligence (AI)-based markers to capture infiltrative heterogeneity using the extracellular water-based voxel-wise characterization of tissue in the peritumoral region.

By leveraging the differences in water diffusivity properties in the peritumoral region of brain metastases and glioblastomas (Central Nervous System (CNS) World Health Organization (WHO) grade 4, Isocitrate-Dehydrogenase 1 (*IDH1)*-wildtype); consisting of purely vasogenic versus infiltrative edema respectively, we train a deep learning model to derive a novel voxel-wise peritumoral microenvironment index (PMI) without using any manual delineation of infiltrative regions. The PMI exploits characterization of water movement restriction in the voxels with and without infiltration and hence captures the infiltrative heterogeneity in the peritumoral region. We later apply connected component analysis to extract locoregional hubs of uniformly high PMI values, hypothesizing them as regions with high infiltration. Descriptive characteristics of locoregional hubs are calculated as AI-based markers of infiltrative heterogeneity, including number and size of hubs and the differences in their shape, direction, and spatial location.

The proposed AI-based markers are utilized for two clinical applications on a population of adult-type diffuse gliomas (CNS WHO grade 4) to demonstrate their potential in capturing distinct locoregional aspects of infiltrative heterogeneity beyond standard diffusion measures: (1) analysis of the duration of survival among *IDH1*-wildtype glioblastomas, to investigate whether higher infiltration of cancer cells, as measured by the proposed markers, is associated with shorter survival; and (2) differences across patients with varying *IDH1* mutation status (astrocytoma *IDH1*-mutant versus glioblastoma *IDH1*-wildtype), notably, the *IDH1*-wildtype tumors, when compared with *IDH1*-mutants, have poorer prognosis, and higher peritumoral infiltration^17,18^.

## Materials and methods

**Figure 1:**
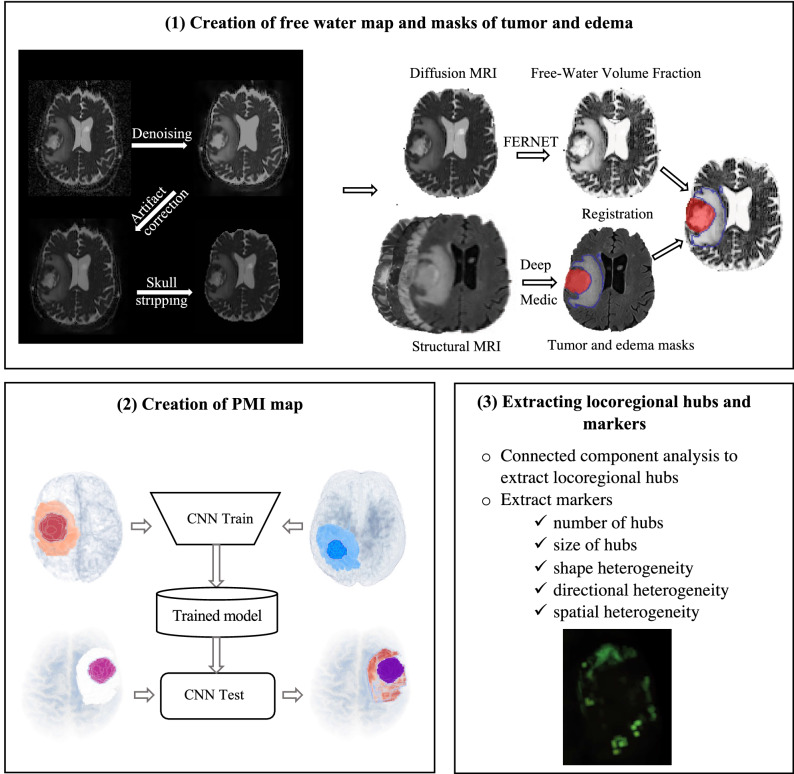
Different steps of the processing pipeline. The pipeline consists of three steps. (**1**) creation of free water map and masks of tumor and edema, (**2**) creation of the PMI map, (**3**) extracting locoregional hubs and AI-based markers. PMI: Peritumoral microenvironment index, CNN: Convolutional neural networks, FERNET: Freewater estimatoR using Interpolated Initialization, MRI: Magnetic resonance imaging.

In this section, we describe the details of the datasets and steps of the pipeline for extracting the proposed AI-based markers. The pipeline consists of three steps. *(i)* creation of free water map and masks of tumor and edema, *(ii)* creation of the PMI map, *(iii)* extracting locoregional hubs and AI-based markers. Figure 1 demonstrates the overview of the pipeline. The proposed markers are evaluated on two clinical applications to investigate whether the differences in infiltrative heterogeneity are able to capture differences in overall survival and *IDH1* mutation status in adult-type diffuse glioma patients.

### Datasets

**Figure 2:**
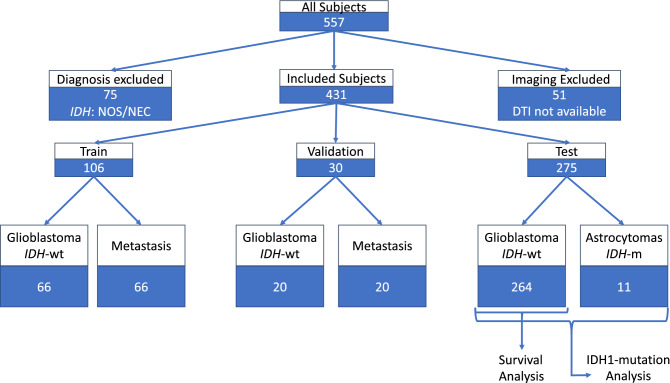
Datasets: train, validation, test (survival and *IDH1*-mutation analysis). *IDH1:* Isocitrate-Dehydrogenase. NOS/NEC: Not elsewhere classified/Not otherwise specified.

This study was approved by the institutional review board of the University of Pennsylvania. Informed consent was obtained from all participants or their legally authorized representative. All methods were carried out in accordance with relevant guidelines and regulations. The population was identified based on retrospective review of the electronic medical record of patients diagnosed with adult-type diffuse glioma (CNS WHO grade 4). Study inclusion criteria were *(i)* histopathological tissue diagnosis of adult-type diffuse glioma (CNS WHO grade 4) or brain metastasis and preoperative; *(ii)* availability of structural sequences (pre (T1) and post-contrast T1 weighted (T1CE), T2 weighted (T2), and T2 weighted fluid attenuated inversion recovery (T2-FLAIR)) and diffusion MRI at time of diagnosis. For the *IDH1* mutation study, sufficient tumor tissue collected at time of surgery was required. We identified 381 patients with adult-type diffuse gliomas (CNS WHO grade 4) and 50 patients with brain metastases, and they were randomly divided into three datasets. Figure 2 demonstrates an overall view of the datasets. **The training dataset** included 106 patients with brain tumors, 66 *IDH1*-wildtype glioblastomas, aged 23–83 years (mean: 60.5, standard deviation (SD): 11.8), and in a male: female proportion of 37:29; and 40 metastases, aged 29–87 years (mean: 62.12, *SD*: 12.6), and in a male: female proportion of 18:22. **The validation dataset** was used to make locoregional hubs. This dataset was independent from the training dataset and consisted of 30 patients with brain tumors, 20 *IDH1*-wildtype glioblastomas and 10 brain metastases, aged 42–84 years (mean: 64.3, SD: 10.5), and in a male: female proportion of 16:14. **The test dataset** was used for two different applications, comprising survival among *IDH1*-wildtype glioblastomas and differences among *IDH1* mutation status. **The overall survival dataset** included 264 *IDH1*-wildtype glioblastomas in the survival range of 0.43 to 76.9 month, aged 21–88 years (mean: 63.6, SD: 11.4), and in a male: female proportion of 160:104. **The mutation dataset** consisted of 275 CNS WHO grade 4 adult-type diffuse gliomas, 264 patients with *IDH1*-wildtype glioblastoma as in the survival data set and 11 *IDH1*-mutant astrocytomas, aged 21–88 years (mean: 62.5, SD: 12.5), and in a male: female proportion of 165:110. The acquisition parameters for all datasets were based on single-shell diffusion data on two Siemens 3 T scanners, Verio or TrioTim, with TR/TE in range of 4200–7400 ms/83–88 ms, with 1 unweighted volume and either 30 or 12-direction diffusion-weighted volumes, at a *b* value of 1000 s/mm^2^. The acquisition was repeated between 1 and 6 times for improved signal to noise ratio (SNR). The spatial resolution was 1.7 × 1.7 × 3 mm.

### Creation of free water map and masks of tumor and edema

All diffusion data was pre-processed using local Principal Component Analysis (PCA) denoising^19^, eddy current and motion correction using FSL EDDY^20^, and skull-stripping with Brain Extraction Tool (BET)^21^. Fractional anisotropy (FA) and mean diffusivity (MD) maps were calculated after DTI fitting with DIPY using weighted least squares^22^.

Structural scans (T1CE, T2, and T2-FLAIR) were registered to the pre-contrast T1 with rigid registration in Advanced Normalization Tools (ANTs)^23,24^, and then registered to the FA image with a nonlinear registration to account for Echo Planar Imaging (EPI) distortions in the diffusion data in ANTs. Masks of the tumor and edema for each patient were created from the registered structural images using Deep-Medic^25^. We used Freewater EstimatoR using Interpolated Initialization (FERNET)^13^, a free water elimination paradigm designed for single-shell diffusion MRI data using a novel interpolated initialization approach, to estimate the free water compartment in single-shell diffusion MRI data. FERNET provides the user with a free water volume fraction map that were resampled to 2×2×2 mm spatial resolution.

### Creation of the PMI map

**Figure 3:**
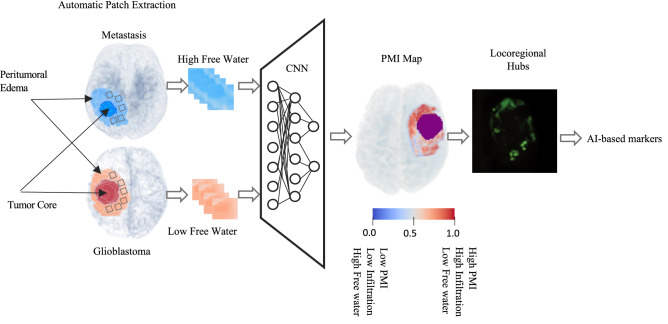
Generation of the PMI map and locoregional hubs. The inputs to the CNN are patches (boxes) extracted from the free water volume fraction map in the peritumoral region from both glioblastomas (red) and metastases (blue) labeled as low free water and high free water which are used to train the CNN. Locoregional hubs are extracted from PMI. Descriptive characteristics of the locoregional hubs are extracted as AI-based markers. PMI: Peritumoral microenvironment index, CNN: Convolutional neural networks.

We used the training dataset to train a convolutional neural network (CNN)^26^ model using the differences in water diffusivity properties of metastasis and glioblastomas. Figure 3 demonstrates a schematic view for the generation of the PMI map. We automatically extracted a set of (16 × 16) voxel patches from the peritumoral edema of metastases and *IDH1*-wildtype glioblastomas placed at random locations and random directions (within axial, sagittal and coronal planes) using random seed generators. This was the largest patch that could be fit into edema without overlapping into the main tumor mass. Patches in the peritumoral edema of metastases and glioblastomas were labeled as high free water and low free water, respectively. The convolutional neural network consisted of 6 convolutional layers followed by a max-pooling and a fully connected layer. A softmax layer at the end produced a probability value for every input patch that indicated its membership to each class, either high free water or low free water^27^. Data augmentation was done on the patches by shifting them in random directions, letting a maximum of 20% overlap with the healthy brain. The hyper-parameters of CNN were weight decay 5 × 10^−5^, momentum 0.9, initial learning rate 10^−4^. The details of the CNN architecture can be found in supplementary material S.1.

To obtain the PMI map for new unseen patients, we placed patches centered at each voxel within the sagittal, axial, and coronal directions. The PMI value for each voxel was then calculated by averaging among the result of the CNN for the patches.

### Extracting locoregional hubs and AI-based markers

**Figure 4:**
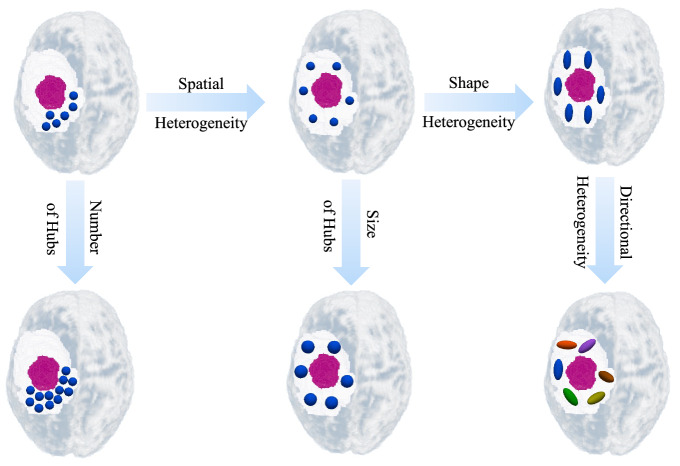
A schematic view of locoregional hubs descriptive characteristics: number of hubs, size of hubs, shape heterogeneity, directional heterogeneity, and spatial heterogeneity. The purple circle displays the tumor core and blue hubs are located in the peritumoral edema. Arrows are directed toward increasing the values of the descriptive characteristics. Figure in the top-left is the reference figure for the hubs. (**i**) Moving from left to right, to the top-middle: the spatial heterogeneity of hubs increases; to the top-right: the shape heterogeneity increases. (**ii**) Moving from top to the bottom, left: the number of hubs increases; center: size of hubs increases; right: directional heterogeneity increases.

For extracting locoregional hubs, we used the validation dataset. Connected components (CCs) of high PMI values^28^ were extracted and defined as locoregional hubs. Descriptive characteristics of locoregional hubs were extracted as AI-based markers for each patient. These characteristics comprised the number and size of the hubs, as well as the differences in their *(i)* shape (quantified by their individual anisotropic property), *(ii)* directionality, and *(iii)* spatial location (see Fig. 4 for a schematic view).

CCs were created for patients in different thresholds in range of [0 1] by steps of 0.1. Connected components with diameters less than 2 voxels were not taken into consideration. The threshold which provided the most significant differences in the descriptive characteristics between patients of the validation data set (30 samples of metastases and glioblastomas) was chosen (threshold = 0.9) and used for the mutation and the survival analysis.

For obtaining descriptive characteristics as AI-based markers, the *number* and *size* of the locoregional hubs were extracted and normalized based on the number of voxels in edema. The size was averaged among the hubs of each patient. Shape heterogeneity and directional heterogeneity were computed as follows: For each locoregional hub, coordinates for all its voxels along three (axial, sagittal, coronal) directions were extracted. Standard deviation of all voxels coordinate values along those three directions were calculated to make a three directional feature vector called sdvec. *Shape heterogeneity* was defined as the normalized difference between the maximum and second largest sdvec, which was later averaged among hubs of each patient. For *directional heterogeneity*, Hausdorff distance^29^ was applied on the pair-wise cosine distance among sdvec of different hubs. For computing *spatial heterogeneity*, the center of gravity for each hub and the average Euclidean distance among them was calculated. This distance was divided by the diameter, or maximum possible distance between any two points to make a normalized value between 0 and 1. A prototype code for extracting AI-based markers is provided in supplementary material S.2.

### Analysis of survival and *IDH1* mutation

The PMI maps were generated for the patients in the survival and mutation datasets. 8 patients were removed from both datasets as they had small edema size (less than two voxels around tumor). Next, the locoregional hubs of high PMI values were generated. 9 patients from the survival dataset and 10 patients from the mutation dataset were removed as they had less than 2 hubs which was the minimum number needed to compute directional and shape heterogeneity.

Survival analysis was done in two parts. First, we divided the population into short- and long-survival groups at the median and analyzed their statistical differences. This was done by doing: *(i)* two sample two tailed *t* test for each of the markers, followed by Bonferroni correction for multiple comparison; and *(ii)* linear regression on each of the markers with sex and age as covariates. Second, clusters of patients were generated downstream of the integration of all markers using K-means. We tried different number of clusters in the range of 1 to 9 and the one that maximized the Calinski-Harabasz index^30^ was chosen. Two clusters were derived, namely, low-PMI and high-PMI patients. Kaplan-Meier (KM)^6^ curves were fit for low-PMI and high-PMI clusters using nonparametric Turnbull estimator^31^. Logrank test and Cox hazard ratio were applied to compare KM-curves. The correlation between different markers were also calculated to explore their similarities.

*IDH1* mutation analysis was done by statistical group difference in two different ways: *(i)* to consider differences in the number of samples in each group, Wilcoxon rank sum^32^ test was done for each of the markers, followed by Bonferroni correction for multiple comparison; *(ii)* linear regression was performed on each of the markers with sex and age as covariates.

## Results

The proposed AI-based markers were evaluated on two clinical applications to investigate whether the differences in infiltrative heterogeneity, as captured by the proposed descriptive characteristics, were able to capture differences overall survival and *IDH1* mutation status of adult-type diffuse glioma patients.

### Survival analysis in *IDH1*-wildtype grade 4 gliomas

**Figure 5:**
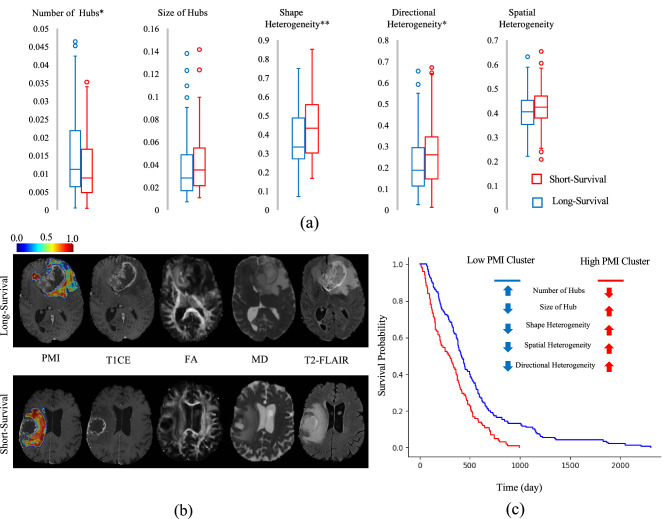
The PMI map and AI-based markers for *IDH1*-wildtype grade 4 glioma patients with different duration of survival. (a) AI-based markers (Descriptive characteristics of PMI locoregional hubs) for long and short survival groups, *p* value < 0.05 (*), *p* value < 0.005 (**), Linear regression was used with age and sex as covariates. (**b**) representative samples of the PMI map with T1CE, FA, MD, and T2-FLAIR images for long and short survival patients. (**c**) Kaplan–Meier estimates of the two clusters generated by K-means clustering. PMI: Peritumoral Microenvironment Index, *IDH1:* Isocitrate-Dehydrogenase 1, T1CE: T1 post-contrast, FA: Fractional anisotropy, MD: Mean diffusivity, T2-FLAIR: T2 weighted fluid attenuated inversion recovery.

The survival analysis was done on a population of 264 *IDH1*-wildtype grade 4 glioma patients in the survival range of 0.43 to 76.9 months. The goal was to determine whether the proposed markers contain information pertaining to patient survival. Statistical analysis between short and long-survival groups demonstrated significant differences in the proposed markers, using *t* test and Bonferroni correction for multiple comparison. The short-survival group had significantly lower number of locoregional hubs (*t*_number_ = 2.54, *p*_number_ = 0.01). Shape and directional heterogeneity were also significantly higher in the short-survival group (*t*_shape_ = 3.89, *p*_shape_ < 0.001, *t*_directional_ = 2.79, *p*_directional_ = 0.005). Linear regression analysis of the markers using sex and age as covariates revealed significant differences between short and long-survival groups (Fig. 5a). The short-survival group had significantly lower number of locoregional hubs (*t*_number_ = 2.65, *p*_number_ = 0.008). Shape and directional heterogeneity were significantly higher in the short-survival group (*t*_shape_ = 3.74, *p*_shape_ < 0.001, *t*_*directional*_ = 2.44, *p*_directional_ = 0.015). Size and spatial heterogeneity were not found to be significantly different. Examples of PMI maps (overlaid with structural MRI), along with T1CE, FA, MD, and T2-FLAIR images, for a short and a long-survival patient are presented in Fig. 5b. KM estimates^6^ of the low-and high-PMI groups of patients (generated using K-means clustering on integration of all markers) are provided in Fig. 5c. A logrank test showed a significant difference between KM curves of low- and high-PMI clusters (*t* = 19.9, *p* < 10^−5^), and the Cox hazard ratio was 1.82 (95% confidence interval: 1.39, 2.37; *p* < 0.005). Group differences among high- and low-PMI clusters, were similar to short and long-survival groups, respectively (Fig. 5c for details and further information in S.3). The correlations between the proposed markers showed the highest correlations between shape heterogeneity and directional heterogeneity. However, they still provide complementary information as coefficient of determination (*R*^2^) showed that only 43% of information in directional heterogeneity was covered by shape heterogeneity (details in S.4).

### *IDH1* mutation analysis of grade 4 gliomas

**Figure 6:**
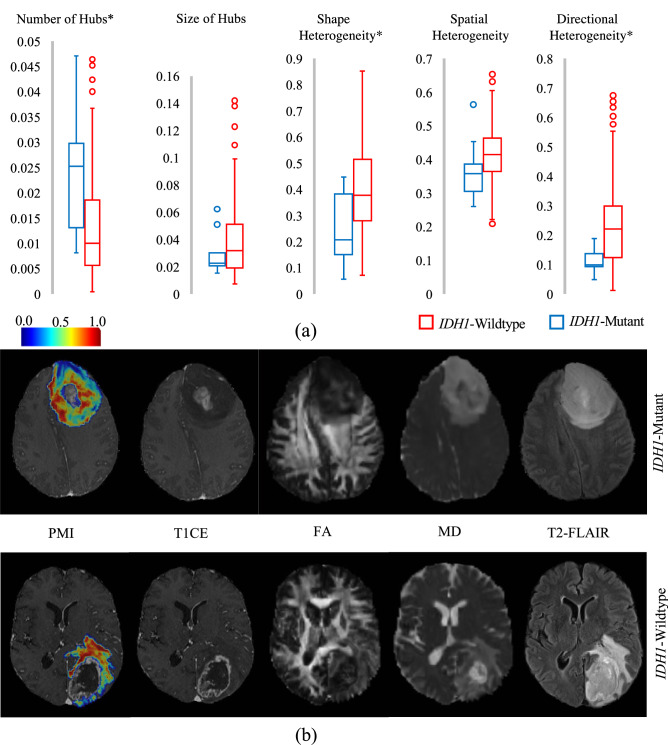
The PMI map and AI-based markers for grade 4 glioma patients with different *IDH1* mutation status. (**a**) AI-based markers (Descriptive characteristics of PMI locoregional hubs) for *IDH1*-mutant and *IDH1*-wildtype groups, *p* value < 0.05 (*), *p* value < 0.005 (**), Linear regression was used with age and sex as covariates. (**b**) representative samples of the PMI map with T1CE, FA, MD, and T2-FLAIR images for *IDH1*-mutant and *IDH1*-wildtype patients. *IDH1*: Isocitrate-Dehydrogenase 1, T1CE: T1- and post-contrast, FA: Fractional anisotropy, MD: Mean diffusivity, T2-FLAIR: T2 weighted fluid attenuated inversion recovery.

*IDH1* mutation analysis was done on a population of 275 CNS WHO grade 4 adult-type diffuse gliomas with different *IDH1* mutation status (i.e., 264 *IDH1*-wildtype glioblastomas vs. 11 *IDH1*-mutant astrocytomas) to investigate whether the proposed markers can characterize mutation status. Statistical analysis across the population, using Wilcoxon rank sum test and Bonferroni correction for multiple comparison, demonstrated significant differences in the proposed markers between *IDH1*-mutants and *IDH1*-wildtypes. The *IDH1*-wildtype group had significantly lower number of locoregional hubs (*z*_number_ = 4.11, *p*_number_ < 10^−4^). Shape heterogeneity and directional heterogeneity were also significantly higher in *IDH1*-wildtypes (*z*_shape_ = 3.15, *p*_shape_ = 0.001, *z*_directional_ = 3.5, *p*_directional_ < 10^−3^). Linear regression analysis of the proposed markers using sex and age as covariates revealed significant differences between the two groups (Fig. 6a). *IDH1*-wildtypes had significantly lower number of locoregional hubs, when compared with *IDH1*-mutants (*t*_number_ = 2.861, *p*_number_ = 0.005). Shape heterogeneity and directional heterogeneity were significantly higher in *IDH1*-wildtypes comparing to *IDH1*-mutants (*t*_shape_ = 2.407, *p*_shape_ = 0.01, *t*_directional_ = 2.380, *p*_directional_ = 0.01). Size and spatial heterogeneity were not found to be significantly different. Examples of PMI maps (overlaid with structural MRI), along with the corresponding T1CE, FA, MD, and T2-FLAIR images for two patients (*IDH1-*mutant astrocytoma and *IDH1*-wildtype glioblastoma) are presented in Fig. 6(b). AI-based markers of the complete population of *IDH1*-mutants versus *IDH1*-wildtype long-survival group versus *IDH1*-wildtype short-survival group are provided in S.5.

## Discussion

We introduced fully automated, novel AI-based markers of the peritumoral microenvironment (PME) of adult-type diffuse gliomas using unexplored information of water restriction extracted from DTI. The markers were based on descriptive characteristics derived from locoregional hubs of the PMI map that captured a unique aspect of the infiltrative heterogeneity from the extracellular water properties of the peritumoral regions of metastases and glioblastomas. Our findings identified a panel of markers as surrogates of infiltration, that could be considered as potential biomarkers of prognosis based on survival and *IDH1* mutation status.

Previous methods on characterizing the infiltrative heterogeneity utilized various imaging modalities using manual or heuristic-based labeling of infiltrative tissue^6–12^. DTI, the modality to characterize water movement, has been used for defining infiltrative heterogeneity in the peritumoral region^33^. However, the extracellular free water movement differences have not been explored for peritumoral region. Therefore, the proposed AI-based markers presented here quantified the heterogeneity in the PME which could complement and further strengthen other diffusion-based measures and MRI parameters to aid in personalized treatment planning toward optimizing patient outcome.

Peritumoral edema, happens due to excess accumulation of fluid in the brain parenchyma which results from infiltrating tumor cells as well as biological responses to the permeability of the spatially adjacent tumor cells^6–8^. The spatial distribution and level of water dynamics pattern in edema has not been well defined yet^6–8^. Using a CNN model trained by characteristics of water diffusivity in glioblastomas versus brain tumor metastases, we were able to capture peritumoral heterogeneity in adult-type diffuse gliomas, without using any manual labeling, to capture surrogates of infiltration and learn how brain parenchyma and immunes system responded to different malignancies.

Brain peritumoral tissue is a heterogeneous microenvironment and accurately quantifying this heterogeneity can be crucial for understanding tumor progression. The proposed markers described regionally distinct water movement centers that contained voxels sharing the same brain tissue microstructures and we demonstrated their utility in two clinical applications.

Previous studies showed that higher infiltration of tumor cells is associated with poorer prognosis^6,7,34,35^. The PMI maps also demonstrated higher PMI values in the voxels of the short-survival patients. Previous literature reporting differences in the levels of FA and MD in altered infiltrated tissues which might be related to higher cellularity and lower water content^7,8,36,37^. Our result provides complementary information in regard to extracellular water movement restriction differences. Analysis of the proposed markers between poor prognosis (short-survivors and high-PMI cluster) and good prognosis (long-survivors and low-PMI cluster), showed a significantly lower number of locoregional hubs in the poor prognosis group which implies larger hubs corresponding to a more infiltrative and aggressive type of tumor. A significantly higher value of shape heterogeneity in the poor prognosis group could reflect the fact that in adult-type diffuse gliomas, cancer cells mainly infiltrate along the white matter^38–40^. Likewise, higher value of directional heterogeneity for poor prognosis patients could reflect the fact that cancer spreads in different directions comparing to good prognosis group. This is consistent with previous literature showing that glioma cells infiltrate brain tissue by at least two topographic paths, including perivascular invasion along the vascular system or infiltration along the extracellular matrix, nerve, and astrocytic tracts^41^.

*IDH1*-wildtype glioblastoma has a poorer prognosis than the *IDH1*-mutant astrocytoma^17,18,34^ which shows higher PMI values. The statistical differences of the proposed markers between *IDH1*-mutants and *IDH1*-wildtypes suggests them as powerful markers to discriminate mutation status, even prior to biopsy. *IDH1*-wildtypes had a lower number of locoregional hubs, which could reflect bigger size of adjacent tumoral cells compared to *IDH1*-mutants. This might represent extracellular vesicles which are involved in the rich network of intercellular connections and develop pathologic cascade leading to neurological diseases^42^. Likewise, higher shape and directional heterogeneity in *IDH1*-wildtypes, could demonstrate that in *IDH1*-wildtypes, cancer spreads more throughout the surrounding brain tissue, reflecting poorer prognosis which might result in higher tumor cellularity and infiltration, causing FA and MD changes^43,44^.

The locoregional hubs-based markers introduced here capture the heterogeneity in masses of adjacent voxels with high infiltration which could correspond to a connected set of glioma cells in the peritumoral region. Multicellular networks with filamentous microtubes are found to connect tumoral cells to a network in the peritumoral space in animal models^41,45^. These cellular networks allow rapid progression and are likely related to treatment resistance^41,45^. Therefore, biologically applicable markers can be identified in highly connected tumor cells that provide insights into the tumor microenvironment and offer translation to the clinic^41,45^.

The proposed markers could be interpreted as signatures of connected tumoral cells. Size and number of hubs quantitated modularity which is a measure of the structure of networks. Lower number of hubs in the poor prognosis group demonstrated their higher modularity. Higher values of shape and directional heterogeneity in poor prognosis group suggest connected tumoral cells with higher shape and directional heterogeneity. This might provide potentially important biomarkers that could be further explored as key targets for treatment planning and patient selection for clinical trials, including novel immunotherapy and anti-invasion therapy^41,45,46^.

The data in this study was collected from a single institution. This limitation was addressed by utilizing different acquisition settings and use of an independent cohort for testing, which provides strong sign of a generalizability of our results. The number of *IDH1* mutant subjects were limited in our study since only approximately 5.6% of primary glioblastomas are *IDH1* mutant^47^. The statistical significance of our findings can be improved by higher number of subjects. Our markers can be extracted fully automatic and do not require any manual intervention or reference labels and are easily translatable to the clinic. Although the proposed markers without a corresponding resected specimen cannot pathologically prove the infiltrative heterogeneity of the peritumoral region, they can be used as surrogate markers for prognosis.

In the future, the proposed markers could be integrated with other imaging modalities to provide biologically relevant characterization of the PME to quantitate invasion and microenvironmental heterogeneity for effective cancer therapy^48–50^. Further studies using the markers described here could be useful to elucidate biological processes linked to the *IDH1* mutation status and better understand multiple sources of heterogeneity in adult-type diffuse gliomas which has major clinical implications^51–53^.

## Supplementary Information

Supplementary Information.

## Data Availability

The datasets generated during the current study are not publicly available due to the IRB requirements of the Hospital of University of Pennsylvania. The datasets can be made available on request to the corresponding author, after required data transfer and IRB paperwork is completed. The code is available here: https://github.com/zrsamani/AI-Based-Locoregional-Markers.
