# Supplementary material for: Artificial intelligence-based locoregional markers of brain peritumoral microenvironment
Source: Sci Rep. 2023 Jan 18;13:963. doi: 10.1038/s41598-022-26448-9 (PMC9849348; doi:10.1038/s41598-022-26448-9)
Supplement: Supplementary file 1 — Supplementary Information. [file 41598_2022_26448_MOESM1_ESM.pdf]

Artificial intelligence-based locoregional markers of brain peritumoral microenvironment

Zahra Riahi Samani*[1,3], Drew Parker[1,3], Hamed Akbari[2,3], Spyridon Bakas[2,3,5], Ronald L. Wolf[3], Steven Brem[4] and Ragini Verma[1,3]

1. Diffusion & Connectomics In Precision Healthcare Research (DiCIPHR) Lab, University of Pennsylvania, Philadelphia, PA 19104, USA; zari@upenn.edu, william.parker@pennmedicine.upenn.edu; ragini@pennmedicine.upenn.edu
2. Center for Biomedical Image Computing and Analytics (CBICA), University of Pennsylvania, Philadelphia, PA 19104, USA; akbariha@upenn.edu; spyridon.bakas@pennmedicine.upenn.edu
3. Department of Radiology, Perelman School of Medicine, University of Pennsylvania, Philadelphia, Pennsylvania, PA 19104, USA; ronald.wolf@pennmedicine.upenn.edu
4. Department of Neurosurgery, Perelman School of Medicine, University of Pennsylvania, Philadelphia, Pennsylvania, PA 19104, USA; steven.brem@pennmedicine.upenn.edu
5. Department of Pathology & Laboratory Medicine, Perelman School of Medicine, University of Pennsylvania, Philadelphia, PA 19104, USA.

Corresponding Author: Zahra Riahi Samani, email: zari@upenn.edu

Supplementary Materials

S.1. CNN architecture and code

The convolutional neural network consisted of 6 convolutional layers followed by a max-pooling and two fully connected layers. A softmax layer at the end produced a probability value for every input patch that indicated its membership to each class, either high free water or low free water. The hyper-parameters were, weight decay: 5×10^{-4}, momentum: 0.9, initial learning rate: 1e-4. We extracted (16,16) patches in the peritumoral area of metastases and glioblastomas and labeled them as high-free water and low-free water, respectively. The patches were input to the CNN, with (3*3) kernels and channel size of (32, 64, 128), followed by a max pooling layer and a flatten layer. As our patch size was small, we did not add more layers and we only used one pooling layer. The output of flatten layer were fed into two dense layers of (512, 2) and last layer produced the membership to each class

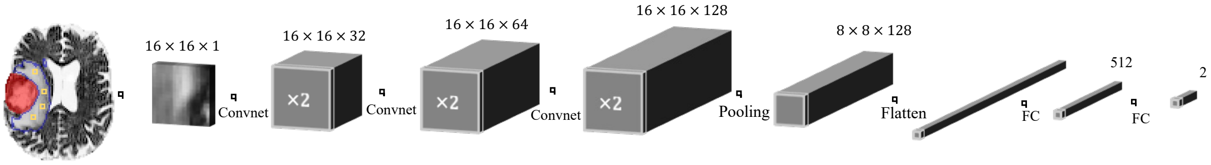


```
from tensorflow.keras.models import Sequential
from tensorflow.keras.layers import Conv2D, MaxPooling2D, Flatten, Dense, Dropout
from tensorflow.keras import optimizers

l1,l2,l3=16,16,1
nClasses = 2

def createModel():
    model = Sequential()
    model.add(Conv2D(32, (3, 3), padding='same', activation='relu', input_shape=(l1, l2, l3)))
    model.add(Conv2D(32, (3, 3), padding='same', activation='relu'))
    model.add(Dropout(0.25))

    model.add(Conv2D(64, (3, 3), padding='same', activation='relu'))
    model.add(Conv2D(64, (3, 3), padding='same', activation='relu'))
    model.add(Dropout(0.25))

    model.add(Conv2D(128, (3, 3), padding='same', activation='relu'))
    model.add(Conv2D(128, (3, 3), padding='same', activation='relu'))
    model.add(MaxPooling2D(pool_size=(2, 2)))
    model.add(Dropout(0.25))

    model.add(Flatten())
    model.add(Dense(512, activation='relu'))
    model.add(Dropout(0.5))
    model.add(Dense(nClasses, activation='softmax'))
    model.compile(optimizer=optimizers.RMSprop(lr=1e-4, momentum=0.9, decay=5e-4),
loss='categorical_crossentropy',
                  metrics=['accuracy'])

    return model

model = createModel()
print(model.summary())
```

S.2. Prototype code for extracting AI-based locoregional markers

Function Marker Extractor ()

Input: connected_components, subject_edema

Output: AI-based locoregional markers including number_of_hubs, size_of_hubs, shape_heterogeneity, directional_heterogeneity, spatial_heterogeneity

```
---- number of hubs
number_of_hubs= number of connected_components

---- computing necessary features
shape_feature, size_feature, spatial_feature, hubs_sd = empty
for i in range (number_of_hubs)
    hubs[i]=connected_components[i]
    size_feature.append (number of voxels in hubs[i])
    xx, yy, zz = coordinates where PMI_map is equal to hubs[i]
    sdvec = [standard_deviation (xx), standard_deviation (yy), standard_deviation (zz)]
    hubs_sd. append(sdvec) ----use for directional_heterogeneity
    sdvec _sorted= sorted (sdvec in decreasing order)
    shape_ feature. append ((sdvec _sorted [0]- sdvec _sorted [1])/ sdvec _sorted [0]) ---use for shape heterogeneity
    xx_mean, yy_mean, zz_mean = mean(xx), mean(yy), mean(zz)
    spatial_feature.append([xx_mean, yy_mean, zz_mean]) ---- use for spatial heterogeneity

---- size and shape heterogeneity
shape_heterogeneity=mean (shape_ feature)
size_of_hubs=mean(size_feature) divided by number of voxels in subject_edema

----directional_heterogeneity
min_Hausdorff =empty
for j in range (number_of_hubs)
     pair_cos_dis=empty
     for k in range (j)
          pair_cos_dis.append (Cosine_distance (hubs_sd[j], hubs_sd[k]))
    min_Hausdorff.append(min( pair_cos_dis))
directional_heterogeneity=max(min_Hausdorff))

----spatial heterogeneity
pair_euclidean_dis=empty
for i in range (number_of_hubs)
    for j in range (i)
          pair_euclidean_dis. append (Euclidean_distance (spatial_feature [i], spatial_feature [j]) divided by diameter
of subject_edema
spatial_heterogeneity=mean(pair_euclidean_dist)
```

S.3. AI-based markers of low-PMI and high-PMI clusters

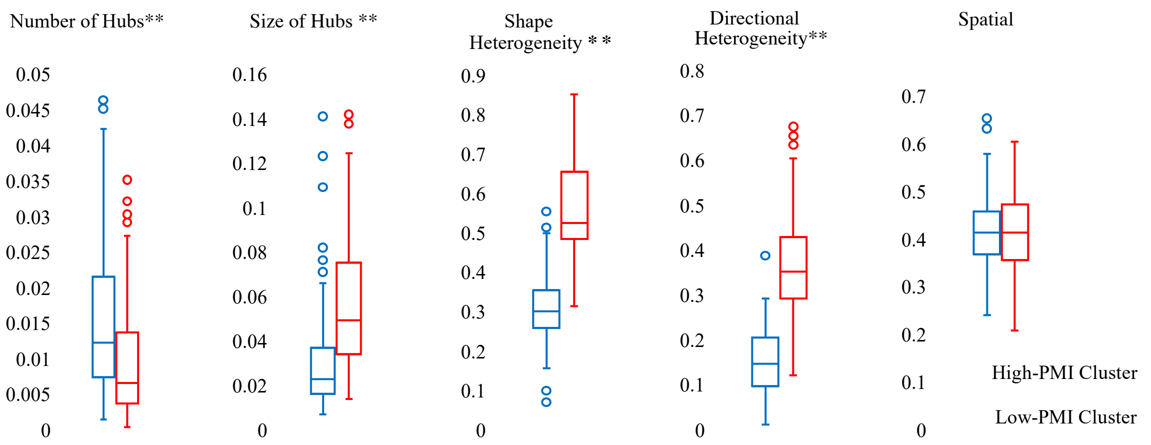

Figure S.3. AI-based markers of PMI locoregional hubs for low-PMI and high-PMI clusters (t-test, p value <0.05 (*), p value<10^{-4} (**)) (PMI: Peritumoral Microenvironment Index).

S.4. Correlations between the proposed AI-based markers

	Number of Hubs	Size of Hubs	Shape Heterogeneity	Directional Heterogeneity	Spatial Heterogeneity
Number of Hubs	X				
Size of Hubs	-0.34	X			
Shape heterogeneity	-0.14	0.23	X		
Directional Heterogeneity	-0.07	0.21	0.65	X	
Spatial Heterogeneity	0.17	-0.24	0.02	0.01	X

S.5. AI-based markers of *IDH1*-mutant vs *IDH1*-wildtype long-survival vs *IDH1*-wildtype short-survival groups

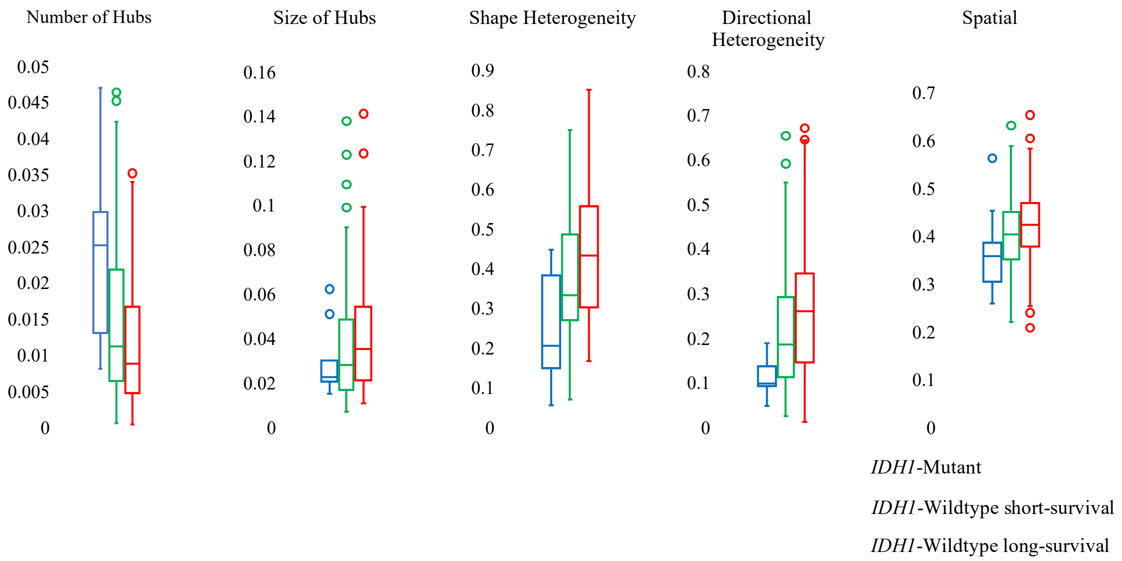

Figure S.5. AI-based markers of *IDH1*-mutant vs *IDH1*-wildtype long-survival vs *IDH1*-wildtype short-survival groups (*IDH1:* Isocitrate-Dehydrogenase 1).
